# Fast gradient algorithm for complex ICA and its application to the MIMO systems

**DOI:** 10.1038/s41598-023-36628-w

**Published:** 2023-07-19

**Authors:** Dariusz Mika

**Affiliations:** The University College of Applied Sciences in Chełm, 22-100 Chełm, Poland

**Keywords:** Engineering, Mathematics and computing

## Abstract

This paper proposes a new gradient-descent algorithm for complex independent component analysis and presents its application to the Multiple-Input Multiple-Output communication systems. Algorithm uses the Lie structure of optimization landscape and toral decomposition of gradient matrix. The theoretical results are validated by computer simulation and compared to several classes of algorithms, gradient descent, quasi-Newton as well as complex JADE. The simulations performed showed excellent results of the algorithm in terms of speed, stability of operation and the quality of separation. A characteristic feature of gradient methods is their quick response to changes in the input signal. The good results of the proposed algorithm indicate potential use in on-line applications.

## Introduction

The area of ​​application of the complex data is currently very wide. It is commonly used in physics, biomedical sciences, wireless communication, sensor array and signal processing etc. For example, most practical modulation methods as phase-shift keying (PSK) and quadrature amplitude modulation (QAM) are of complex type. These types of digital modulation techniques are used in various wireless communication systems. One technique used in this area of ​​communication is the multi-antenna technique, which is used to improve the performance and robustness of wireless links. The Multiple-Input Multiple-Output (MIMO) system uses the multi-antenna technique on both the transmitting and receiving side. The modulated complex signal reaching the receiving antennas is a mixed signal from the transmitting antennas. The recovery of the useful signal requires use of the mixed signal separation techniques. Blind Source Separation (BSS) is a wide range of techniques for recovering the unobserved sources signals from the observed channel output without any quantitative information of the channel system. The BSS methods have solid theoretical foundations and extensive potential applications^1^. An excellent overview of the BSS techniques for interference cancellation in communication systems can be found in^2^. Constant modulus (CM) criterion is probably the best known and most studied higher order statistics based criterion in blind equalization^3^ and signal separation^4,5^ areas. It exploits the idea that certain communication signals have the constant modulus property. The Constant Modulus Algorithm (CMA) was initially designed for PSK signals^6^. In the case of constant modulus signals CMA has proven reasonable performances and desired convergence requirements. On the other hand using the CMA for nonconstant modulus signals such as QAM signals yields degraded performance because the CMA projects all signals points onto a single modulus. In order to improve the performance of CMA for QAM signals a number of modified methods have been introduced such as the algorithm known as MMA^7^ and constant norm algorithm (CNA) ^8^ whose CMA represents a particular case. The CMA can also be used in MIMO systems^9^. However this case is susceptible to the one-to-many problem. The CMA uses the CM feature and it may not identify the desired signal individually when several constant modulus signals coexists and often yield the same signal on the output channel. In order to omit this drawback a lot of extensions for CMA have been introduced. For example Least-squares multi-user CMA^10^, Recursive least-squares CMA^11^, kurtosis-based method (MUT)^12^, CMA using Givens rotation^13^ and Succesive interference cancellation least- squares CMA^14^.

A typical CMA algorithm is to minimize the cost function of the form ${J}_{CM}=E\left\{{\left({\left|y\right|}^{2}-\gamma \right)}^{2}\right\}$, where $\gamma$ is a constellation-dependent parameter. These algorithms are therefore designed for specific digital communication signals, i.e. signals with the same constant modulus $\gamma$. Therefore, in the available literature, the presented simulation experiments are designed for a small number of signals of the same type, e.g. MIMO 2 × 3 system with transmission of only BPSK signals^15^, 4 × 2 system with only PSK(4)^16^ or PSK(8)^17^, QAM(4)^14,18^, QAM(16)^17^ and QAM(64) signals^8^. In our experiment presented in Chapter 8, we used, among others, the MIMO 5 × 8 system with the transmission of the five above-mentioned digital modulation signals at once. The positive results of the experiment testify high flexibility of the proposed algorithm and the cost function used.

In this paper we deal with another very common technique in BSS known as Independent Component Analysis (ICA). This method and its related Independent Vector Analysis (IVA) are widely used in all kinds of areas requiring signal extraction^19^. The ICA method depends on the separation of independent components (i.e., sources) and relies on the assumption of their statistical independence. There are many methods for solving the ICA problem. The earlier methods include algebraic methods in which the demixing matrix is derived algebraically from different matrices containing specific signal statistics. In the FOBI^20^ method, it is the covariance matrix and the matrix based on fourth-order moments, and in the JADE^21^ method (Joint Approximate Diagonalization of the Eigenvalue matrices) it is cumulative matrices. The alternative approach to the ICA problem is based on the search of the extreme of a specially constructed real cost function in the complex domain. The essence of the construction of the cost function consists in its respective non-linearity generating higher-order statistics. The following approaches are used, i.e., as: Maximum Likelihood, Maximization of Mutual Information, Maximization of non-Gausianity and Information-Maximization framework. All these approaches are closely related to each other^22^. The ICA algorithms are primarily optimization-iterative algorithms, the speed of which depends to a large extent on the adopted optimization strategy. The gradient algorithms, also known as the steepest descent, use first-order information about the cost function and are characterized by a linear degree of convergence. In Newton or quasi-Newton algorithms where, in addition to first-order information about the cost function, second-order information is used and the degree of convergence is square or even cubic. These algorithms, due to the need to use elements of the Hessian matrix, are characterized by high computational complexity, but due to the speed of convergence, they are much faster than gradient algorithms. A typical example of this type of algorithm is the so-called a fixed point algorithm called FastICA, which was introduced in the complex version in^23^. The algorithm assumes circular nature of the source signals, i.e., their pdf are symmetrical in the complex plane. A variation of this algorithm for noncircular signals was introduced in^24^.

On the other hand, unlike Newton algorithms, gradient algorithms are characterized by a high speed of response to changes in the input signal, which means that they have a great potential for use in on-line applications. In classical gradient algorithms, the gradient of the cost function is determined in the Euclidean space. In the case of imposing a constraint on the optimization space, as in the case of the classic ICA (unitarity of the demixing matrix), the Lagrangian type of optimization methods by adding extra-penalty term is used, and the gradient is also determined in the Euclidean space. Such an approach causes that in each iteration the constraint condition is lost and the boundary surface is dropped (the demixing matrix loses the unitarity property). After each iteration, a re-unitarization operation is required to restore the constraint condition and return to the optimization surface. This classic approach does not take into account the special structure of the optimization parameter space. If this space is treated as a differential manifold then this constrained optimization problem can be seen from the point of view of Riemann geometry. In this case, the gradient is defined as the vector tangent to the constrain surface. In this case, also in each iteration step the boundary surface is exited, however the optimization movement always takes place in the tangent direction to the constraint surface which affects the speed and stability of the algorithms. Taking into account the algebraic structure (i.e., group structure) of the ICA model parameter space, the constraint condition is maintained in each iteration step. The classical complex ICA model has the structure of the unitary group $\mathrm{U}(n)$, i.e., the optimization space is a group of matrices satisfying the condition $W{W}^{H}={W}^{H}W=I$. This group is closed under the matrix multiplication operation. Thus, when using the multiplicative iteration procedure, the condition of the unitarity of the demixing matrix $W$ is met. The unitary group $\mathrm{U}(n)$ with its smooth differential structure forms the Lie group. The curve that minimizes the path between two points on a Riemannian manifold is called a geodesic carve and is equivalent to a straight line in an ordinary flat Euclidean space. Optimization methods based on this convenient optimization space structure are called geodesic methods and can be found in^25,26^. The optimization motion in this case follows the geodesic curve in each iteration step.

This paper is organized as follows. In section "Background on ICA", a basic model of complex ICA is provided. Various approaches to the ICA problem are presented as well. Section "Lie group and their Lie algebra" is devoted to the general concept of Lie groups and Lie algebras. Riemannian and Lie structure of unitary group, Riemannian gradient, geodesic motion in Lie group of unitary matrices are presented in section "Optimization on unitary group ". The toral geometry in optimization landscape is established in section "Toral geometry in optimization landscape". The computational complexity is studied in section "Computational complexity". The MIMO telecommunication system is described in section "Multiple-Input Multiple-Output systems". The application of the proposed algorithm in the MIMO system and comparison with other ICA algorithms is presented in Chapter 8.

## Background on ICA

The classical linear model of ICA is defined as

1 $$x=As$$

and in noisy case

2 $$x=As+\sigma \vartheta$$

where $x={({x}_{1},\dots ,{x}_{n})}^{T}\in {\mathbb{C}}^{n}$ are only known observed signals, $A\in {\mathbb{C}}^{n\times n}$ is an $n\times n$ complex-valued, unknown, invertible mixing matrix, $s={({s}_{1},\dots ,{s}_{n})}^{T}\in {\mathbb{C}}^{n}$ are unknown complex-valued source signals, i.e., independent components, $\vartheta \sim CN(0,{I}_{n})$ is circular zero-mean Gaussian noise with covariance matrix ${I}_{n}$ and ${\sigma }^{2}$ is the variance of added noise to the mixed signals. Sources ${s}_{i}$ are assumed to be mutually statistically independent. Sources estimates $\widehat{s}={({\widehat{s}}_{1},\dots ,{\widehat{s}}_{n})}^{T}$ are given by inverse model (in the noiseless case)

3 $$\widehat{s}=Wx=WAs$$

where $W\cong {A}^{-1}$ is called demixing matrix.

The aim of ICA is to find a demixing matrix $W$ having only $N$ realizations of the observed signal $x$. Above systems can be solved up to complex scale accuracy (i.e., up to phase and modulus) and permutations of ${s}_{\mathrm{i}}$ provides that at most one sources is Gaussian. The classical ICA model assumes that the number of source signals ${s}_{\mathrm{i}}$ is known and is equal to the number of observed signals ${x}_{\mathrm{i}}$. In the general ICA model, this condition does not have to be met and a case in which the number of source signals is different from the number of observed signals is considered. In the extreme case, when a single observed signal is given, the problem is called Single Channel Source Separation, which is also possible to solve^27–29^.

In order to reduce the computational complexity, the whitening (or sphering) process is usually performed on observed signal in the preprocessing stage. In this stage observed signals are decorrelated using eigendecomposition of correlation matrix. It is assumed, without losing generality, that the sources have a mean value of zero and a unit variance, i.e., $E\left\{s{s}^{H}\right\}=I$. Whitening process is given by $y={\Lambda }^{-1/2}{U}^{H}\mathrm{x}$, where $U$ is matrix of eigenvectors and $\Lambda$ is diagonal matrix with eigenvalues on main diagonal. It is easy to check that whitened signals are decorrelated, i.e., $E\left\{y{y}^{H}\right\}=E\{{\Lambda }^{-1/2}{U}^{H}x{x}^{H}U{\Lambda }^{-1/2}\}={\Lambda }^{-1/2}{U}^{H}E\{x{x}^{H}\}U{\Lambda }^{-1/2}=I$. Denoting $V=U{\Lambda }^{-1/2}$ as the whitening transformation matrix it can be written $y=Vx=VAs=\widetilde{A}s$, where $\widetilde{A}$ is a new mixing matrix with unitary properties $E\left\{y{y}^{H}\right\}=\widetilde{A}E\left\{s{s}^{H}\right\}{\widetilde{A}}^{\mathrm{H}}=\widetilde{A}{\widetilde{A}}^{H}=I$. It can be seen that whitening process simplifies the ICA problem from optimization on general linear group $\mathrm{Gl}\left(n\right)$ (matrix $W$ satisfies only invertibility property $\mathrm{det}(W)\ne 0$) to optimization on unitary group $\mathrm{U}\left(n\right).$

There are different approaches to solve ICA problem. Main two of them are optimization of cost function and algebraic methods. The first class includes methods like: Maximum Likelihood estimation (ML)^30^, Mutual Information Minimization or Information Maximization^31^ and Negentropy Maximization^23^. The second class includes, for example, the JADE and FOBI algorithms ^20^. First class of methods uses nonlinear cost function to archive signal independence. In ML approach the negative log-likelihood function is used as the cost function, which takes the form

4 $$\ell \left(W,{W}^{*}\right)\triangleq -\mathrm{log}p\left(x, {x}^{*}\right)=-\mathrm{log}\left|\mathrm{det}\left(W\right)\mathrm{det}\left({W}^{*}\right)\right|- \sum_{i=1}^{n}\mathrm{log}{p}_{i}({w}_{i}^{H}x, {w}_{i}^{T}{x}^{*})$$

where ${w}_{i}$ denotes i*-*th column of $W$*,* ${p}_{i}(\cdot )$ is the pdf function of *i-*th source random variable ${s}_{i}$. Function $\ell\left(\cdot \right)$ and ${p}_{i}(\cdot )$ are expressed in dual notation as $\ell\left(W,{W}^{*}\right)$ and ${p}_{i}({s}_{i},{s}_{i}^{*})$. Using Wirtinger calculus (see Appendix) and the related matrix differential roles it can be shown^32^ that the gradient matrix takes the form

5 $${\nabla }_{{W}^{*}}\ell=\frac{\partial \ell}{\partial {W}^{*}}=-{W}^{-H}+\varphi \left(Wx\right){x}^{H}$$

where $$\varphi \left(Wx\right)\equiv \varphi \left(s,{s}^{*}\right)={\left({\varphi }_{1}\left({s}_{1}, {s}_{1}^{*}\right),\dots ,{\varphi }_{n}\left({{s}_{n},s}_{n}^{*}\right)\right)}^{T}$$ and

6 $${\varphi }_{i}\left({s}_{i}\right)\triangleq -\frac{\partial \mathrm{log}{p}_{i}({s}_{i},{s}_{i}^{*})}{\partial {s}_{i}^{*}}= -\frac{1}{2}\left(\frac{\partial \mathrm{log}{p}_{i}({s}_{Ri},{s}_{Ii})}{\partial {s}_{Ri}}+i\frac{\partial \mathrm{log}{p}_{i}({s}_{Ri},{s}_{Ii})}{\partial {s}_{Ii}}\right)$$

is the score function or nonlinearity of the *i-*th source variable. Stochastic gradient update rule for $W$ can be written as in (Appendix A6)

7 $$\Delta W=-\mu \frac{\partial \ell}{\partial {W}^{*}}=-\mu \left({W}^{-H}-\varphi \left(Wx\right){x}^{H}\right)$$

Its relative (or natural) version introduced by Amari^33^ takes the form

8 $$\Delta W=-\mu \frac{\partial \ell}{\partial {W}^{*}}{W}^{H}W=-\mu \left(I-\varphi \left(Wx\right){\left(Wx\right)}^{H}\right)\mathrm{W}$$

Assuming $\varphi \left( s \right) = 2\tanh\left( s \right)$, then this update rule is known as the complex Infomax algotithm^31^.

Another very important approach to ICA problem uses some non-Gaussianity measure as the cost function^22^. The natural cost function in that class is negentropy that measure the entropic distance of pdf of the source estimation from Gaussian distribution and can be written as

9 $$J\left(W\right)\triangleq H\left(\nu \right)-H(s)$$

where $H\left(\cdot \right)\triangleq -E\{\mathrm{log} p\left(\cdot \right)\}$ is the differential entropy of the given distribution and $\nu ={\nu }_{R}+i{\nu }_{I}$ is the Gaussian-distributed complex variables with the same variance as source estimate $s$. Since $H\left(\nu \right)$ in (9) has a constant value for a given covariance matrix, maximizing negentropy is equivalent to minimizing the entropy of $H(s)$. The main problem with this approach is that negentropy is computationally very difficult. Definition of negentropy requires knowledge of unknown distribution $p\left(\cdot \right)$. In practical applications, some negentropy approximations based on the idea of Taylor expansion and maximum entropy methods are used. The Gram–Charlier and the Edgeworth expansions are used usually in this context. They lead to similar approximation based on Chebyshev-Hermite polynomials and higher order cumulants. In^22^ there is an excellent introduction to this topic. However, this approach leads sometimes, to rather poor approximation due to the fact that sample estimator of higher-order cumulants are very sensitive to outliers and cumulants themselves measure mainly the tails of the distribution and are not very sensitive to structure near the center of the distribution. To overcome this problem, the maximum entropy and nonpolynomial expansion are used, which in real case leads to approximation of negentropy of the form^22^

10 $$J\left(x\right)\approx \frac{1}{2}\sum_{i=1}^{n}{E\{{G}^{i}\left(x\right)\}}^{2}$$

where ${G}^{i}, i=1,\dots ,n$ is any set of orthonormal nonquadratic function. Nonquadratic assumption results from the need for integrability of the pdf function obtained in the maximum entropy method and more robustness against outliers. A simple special case of this approximation can be obtained using only one nonquadratic function, which in the complex case takes the form

11 $$J\left(W\right)=E\{{\left|G\left(Wx\right)\right|}^{2}\}$$

where $G: {\mathbb{C}}\to {\mathbb{C}}$ is nonquadratic function which matches the source pdf by rather symbolic relation ${p}_{S}\left(s\right)={p}_{S}\left({s}_{R},{s}_{I}\right)=K\mathrm{exp}\left({\left|G\left(s\right)\right|}^{2}\right)$*,* where $K$ is normalizing constant. In classical complex FastICA (cFastICA) algorithm^23^ cost function takes the form

12 $$J\left(W\right)=E\left\{G\left({\left|Wx\right|}^{2}\right)\right\}$$

where in this case nonlinear function $G: {\mathbb{R}}\to {\mathbb{R}}$ is chosen as a smooth even function, i.e., $G\left(u\right)=\frac{1}{2}{u}^{2}$ or $G\left(u\right)=\mathrm{log}(0.1+u)$. It is worth noting that nonlinear function $G: {\mathbb{C}}\to {\mathbb{C}}$ in (11) uses full complex nature of signals, i.e., phase and magnitude. While nonlinear function in (12) does not preserve phase information and uses only signal’s magnitude. Therefore, cFastICA assumes that the sources have a circular distribution and fails in the case of noncircular sources^34^.

Expressing cost function in (11) in dual form as

13 $$J\left(W\right)=E\left\{G\left(s\right)G{\left(s\right)}^{*}\right\}=E\{G\left(s\right)G\left({s}^{*}\right)\}$$

where $s={W}^{H}x$ and using Wirtinger calculus, it is easy to derive the gradient of this cost function in the form

14 $${\nabla }_{{W}^{*}}J=\frac{\partial J}{\partial {W}^{*}}=E\left\{xG\left({W}^{T}{x}^{*}\right){G}^{\mathrm{^{\prime}}}\left({W}^{H}x\right)\right\}=E\{xG(s{)}^{*}{G}^{\mathrm{^{\prime}}}\left(s\right)\}$$

where ${G}^{^{\prime}}\left(s\right)=\frac{dG}{ds}$ is the derivative of $G$, so the gradient descent update rule can be expressed as

15 $$\Delta W=\mu E\{xG(s{)}^{*}{G}^{\mathrm{^{\prime}}}\left(s\right)\}$$

where $\mu$ denotes the step size.

More efficient algorithms use second-order information about cost function. In^23^ cFastICA algorithm was derived using constrained optimization approach and quasi-Newton technique. The cFastICA algorithm update rule takes the form

16 $${W}_{n+1}=-E\{G\left(s{)}^{*}g\left(s\right)x\right\}+E\left\{g\left(s\right){g}^{*}\left(s\right)\right\}{W}_{n}+ E\{x{x}^{T}\}E\{G\left(s{)}^{*}g\mathrm{^{\prime}}\left(s\right)\right\}{W}_{n}^{*}$$

and ${W}_{n+1}$ must be orthogonalized in every iteration.

In algebraic complex ICA methods as FOBI the demixing matrix is determined algebraically by the simultaneous diagonalization of a covariance matrix and the matrix based on fourth-order moments. The JADE algorithm is a generalization of FOBI. Algorithm uses Givens rotations to transform the set of ${n}^{2}$ fourth-order moments to more diagonal form—two rows and two columns at a time. Unfortunately, it is well known that computational cost of JADE grows quickly with the number of components $n$. For a comparison of these two methods see^35^.

## Lie group and their Lie algebra

A Lie group is a topological group $G$ which also has got the properties of a smooth differential manifold of class ${\mathrm{C}}^{\infty }$ where the algebraic and differential structures are compatible, i.e., the mapping $G\times G$ into $G$ given by the formula^36^

17 $$G\times G\ni \left(g,h\right)\to g{h}^{-1}\in G$$

is also of class ${\mathrm{C}}^{\infty }$.

A Lie algebra is the linear space $L$ over the commutative field $K$, for which a bilinear operation is defined

18 $$L\times L\ni \left(a,b\right)\to [a,b]\in L$$

called Lie bracket, satisfying the conditions

- $$\left[a,b\right]=-[b,a]$$ (antisymmetry)
- $$\left[a,\left[b,c\right]\right]+\left[c,\left[a,b\right]\right]+\left[b,\left[c,a\right]\right]=0$$ (Jacobi identity)

The important class of homeomorphic mapping called left and right translation by $w\in G$ are associated with Lie groups and are defined as

19 $${L}_{w}: G\ni g\to wg\in G;w\in G$$

20 $${R}_{w}: G\ni g\to gw\in G;w\in G$$

**Figure 1:**
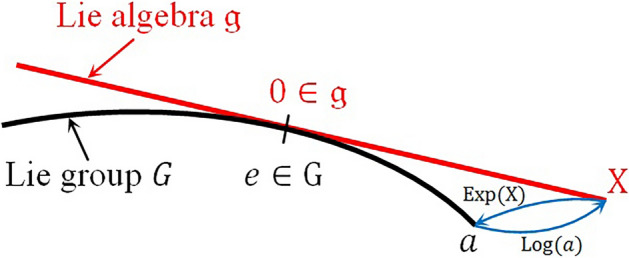
One-dimensional representation of the relationship between the Lie group $G$ and its Lie algebra $\mathfrak{g}$.

Those homeomorphisms are diffeomorphisms inducing tangent mapping

21 $${\left({L}_{w}\right)}_{*}:{\Gamma }^{\infty }\left(G\right)\to {\Gamma }^{\infty }\left(G\right): X\to {\left({L}_{w}\right)}_{*}X$$

22 $${\left({R}_{w}\right)}_{*}:{\Gamma }^{\infty }\left(G\right)\to {\Gamma }^{\infty }\left(G\right): X\to {\left({R}_{w}\right)}_{*}X$$

where ${\Gamma }^{\infty }\left(G\right)$ is the vector field tangent to $G$. Any vector field ${X\in \Gamma }^{\infty }\left(G\right)$ with property ${\left({L}_{w}\right)}_{*}X=X$ or ${\left({R}_{w}\right)}_{*}X=X$ for any $w\in G$ is called left or right invariant. The left and right invariant fields are Lie algebra $\mathfrak{g}$ and this algebra is isomorphic to tangent space ${T}_{e}G$ in the identity element $e$ of the group $G$^36^. A space ${T}_{e}G$ with this Lie structure is called the Lie algebra of the Lie group. This algebra can be used to parameterize the neighborhood of identity element $e$ via application of homeomorphism known widely as the exponential map or simply as exponential denoted as $\mathrm{exp }: \mathfrak{g}\to G$ and given by the expression

23 $$\mathrm{exp}\left(X\right)={\gamma }_{X,e}(1)$$

where ${\gamma }_{X,e}$ is the integral carve of the left or right invariant field $X$ on Lie group $G$ passing through the identity element $e$ and is called as one-parameter subgroup in $G$. The inverse of the exponentiation is called the logarithmic mapping $\mathrm{Log }:\mathrm{G}\to \mathfrak{g}$ and they are locally bijective. Figure 1 shows geometrical relationship between a Lie group and its Lie algebra.

## Optimization on unitary group $U\left( n \right)$

Complex ICA problem can be effectively considered as the optimization on the unitary group $\mathrm{U}(n)$ and applying the tools of Riemannian geometry. Matrix representation of the group $\mathrm{U}\left(n\right)$ is defined as

24 $$\mathrm{U}\left(n\right)=\{W\left|{W\in {\mathbb{C}}^{n\times n}, W}^{H}W=I\}\right.$$

with identity element $e=I$.

The one-parameter subgroup ${\gamma }_{X,I}\left(t\right):t\to \mathrm{U}\left(n\right)$ of matrix representation of $\mathrm{U}\left(n\right)$ satisfies the condition ${{\gamma }_{X,I}{\left(t\right)}^{H}\gamma }_{X,I}\left(t\right)=I$. By differentiating this equation with respect to $t$, the condition defining the tangent space ${T}_{W}\mathrm{U}(n)$ at the point $W$ is obtained and takes the form

25 $${T}_{W}\mathrm{U}\left(n\right)=\{X\in {\mathbb{C}}^{n\times n}\left|{{X}^{H}W+ W}^{H}X=0\}\right.$$

The Lie algebra $\mathfrak{u}\left(n\right)$ (of a Lie group $\mathrm{U}\left(n\right)$) is obtained by substituting $W=I$ in (25)

26 $$\mathfrak{u}\left(n\right)={T}_{I}\mathrm{U}\left(n\right)=\{X\in {\mathbb{C}}^{n\times n}\left|{X}^{H}+X=0\}\right.$$

This algebra is a set of skew-Hermitian matrices.

By defining the inner product, i.e., the metric, the Riemann structure is established on the group $\mathrm{U}\left(n\right)$. Inner product induced from ambient Euclidean space $\mathcal{A}={\mathbb{C}}^{n\times n}$ and its inner product $\langle X,Y{\rangle }_{A}=\mathrm{tr}\left({X}^{H}Y\right)$ obtains the form^37^

27 $${\langle X,Y\rangle }_{W}=\frac{1}{2}\mathrm{tr}\left({X}^{H}Y\right)$$

and determines the canonical bi-invariant metric on $\mathrm{U}\left(n\right)$.

Let $f:\mathrm{U}\left(n\right)\to {\mathbb{R}}$ be a differentiable real-valued function defined on $\mathrm{U}\left(n\right)$. The maximum rate of change $f$ is obtained in the direction of the negative conjugate gradient of the function expressed by rather symbolic form as

28 $${X}_{W}=-{B}_{W}^{-1}{\nabla }_{{W}^{*}}f$$

where ${B}_{W}$ is the Hessian term. In gradient descent algorithms ${B}_{W}$ is the identity matrix $I$, while in Newton or quasi-Newton methods ${B}_{W}$ is exact or some approximation of Hessian tensor. In the Riemannian manifold, a contravariant version of the gradient vector is considered. The conjugate gradient of the cost function ${\nabla }_{{W}^{*}}f$ in this version can be derived from

29 $${\langle {\nabla }_{{W}^{*}}f,X\rangle }_{A}={\langle {\widehat{\nabla }}_{{W}^{*}}f,X\rangle }_{W}$$

where ${\widehat{\nabla }}_{{W}^{*}}f\in {T}_{W}\mathrm{U}\left(n\right)$ is the contravariant version of the sought conjugate gradient ${\nabla }_{{W}^{*}}f\in {T}_{W}\mathcal{A}$ and $X\in {T}_{W}\mathrm{U}\left(n\right)$ is any tangent vector. Taking into account the inner product on an ambient Euclidean space $\mathcal{A}={\mathbb{C}}^{n\times n}$ and on an embedded manifold $\mathrm{U}\left(n\right)$ defined above, it can be shown^38^ that the contravariant gradient of the cost function on Lie group $\mathrm{U}\left(n\right)$ at the point $W$ can be expressed as

30 $${\widehat{\nabla }}_{{W}^{*}}f={\nabla }_{{W}^{*}}f-W{\nabla }_{{W}^{*}}^{\mathrm{H}}fW\in {T}_{W}\mathrm{U}(n)$$

In the Riemann manifold, the curve minimizing length between two points is called geodesic. Geodesic on unitary group of matrices emanating from $I$ in $X$ direction can be expressed using exponential map^36^

31 $${\gamma }_{X,I}\left(t\right)=\mathrm{exp}(tX)\in \mathrm{U}\left(n\right)$$

Exponentiation of $n\times n$ matrices is given here by convergent power series $\mathrm{exp}\left(X\right)={\sum }_{n=1}^{\infty }\frac{{X}^{n}}{n!}$. The left and right translation in (19) and (20) maps geodesic in point $W$ to geodesic in identity $I$ by the relation ${\gamma }_{X,I}\left(t\right)={W}^{-1}{\gamma }_{X,W}\left(t\right)={W}^{H}{\gamma }_{X,W}\left(t\right)$ and ${\gamma }_{X,I}\left(t\right)={\gamma }_{X,W}\left(t\right){W}^{-1}={\gamma }_{X,W}\left(t\right){W}^{H}$, therefore, the geodesic emanating from $W$ has the form

32 $${\gamma }_{X,W}\left(t\right)={W\gamma }_{X,I}\left(t\right)=W\mathrm{exp}(tX)$$

and

33 $${\gamma }_{X,W}\left(t\right)={\gamma }_{X,I}\left(t\right)\mathrm{W}=\mathrm{exp}(tX)W$$

using left and right translation, respectively.

In the case of matrix representation of unitary group $\mathrm{U}(n)$ relationship (32) and (33) can be expressed as

34 $${\gamma }_{X,W}\left(t\right)={W\gamma }_{X,I}\left(t\right)=WR$$

and

35 $${\gamma }_{X,W}\left(t\right)={\gamma }_{X,I}\left(t\right)\mathrm{W}=RW$$

where $R$ is a complex rotation matrix and establishes a local parameterization. Gradient descent algorithms using this type of parameterization are called geodesic (or geodesic flow) methods. In this method the Lie algebra element is mapped exactly to the Lie group elements. The most popular geodesic parameterization used to describe a small neighborhood of the point in $\mathrm{U}(n)$ is, the previously mentioned, exponential that can be expressed using Taylor expansion

36 $$R=I+tX+\frac{{t}^{2}}{2}{X}^{2}+\frac{{t}^{3}}{6}{X}^{3}+\cdots$$

In practice some approximation of exponential are used. An overview of the different types of approximations can be found in^39^. The most efficient of them are: diagonal Páde, Generalized Polar Decomposition, truncated Taylor series. Another popular parameterization method is Cayley transform of a form $R=(I-tX{)}^{-1}(I+tX)$. Using Taylor expansion of $R$ it can be shown that up to the second-order term Cayley transform is equivalent to the matrix exponential^37^.

The idea of the standard geodesic flow minimization procedure is to perform an optimization motion (in the Lie algebra $\mathfrak{u}\left(n\right))$ in the direction of the negative gradient $-{\widehat{\nabla }}_{{W}^{*}}f$ defined in (30) and then map to the Lie group $\mathrm{U}\left(n\right)$ using some kind of local parameterization. The search direction is obtained by translating the contravariant gradient ${\widehat{\nabla }}_{{W}^{*}}f\in {T}_{W}\mathrm{U}\left(n\right)$ to the identity $I$, i.e., into the Lie algebra $\mathfrak{u}\left(n\right)$. Using expression (30) and tangent map (21) the search direction takes the form

37 $${\Omega ={W}^{-1}\widehat{\nabla }}_{{W}^{*}}f={{W}^{H}\widehat{\nabla }}_{{W}^{*}}f={W}^{H}{\nabla }_{{W}^{*}}f-{\nabla }_{{W}^{*}}^{\mathrm{H}}fW\in \mathfrak{u}\left(n\right)$$

which is obviously a skew-Hermitian matrix.

A set of skew-Hermitian matrices ${\mathfrak{u}}_{\Omega }\left(n\right)=\{t\Omega \left|\Omega \in \mathfrak{u}\left(n\right), t\in {\mathbb{R}}\}\right.$ also forms a Lie algebra known as the one-parameter subalgebra of the Lie algebra $\mathfrak{u}\left(\mathrm{n}\right)$. In this case, however, it is the abelian (commutative) algebra associated with one-parameter subgroup ${R}_{\Omega }(t)=\mathrm{exp}(t\Omega )$. The one-parameter optimization methods consist of searching for a minimum of the cost function along the subalgebra ${\mathfrak{u}}_{\Omega }\left(n\right)$, i.e., for a chosen search direction ${\Omega }_{0}$ which corresponds to the search along the subgroup ${R\Omega }_{0}(t)$. After finding the ${t}_{opt}$, i.e., the value of the $t$ parameter for which $f$ reaches the minimum in the ${{W}_{0}R\Omega }_{0}(t)$ subgroup, where ${W}_{0}$ is the starting point, a new search direction ${\Omega }_{1}$ is calculated and the procedure is repeated until convergence. Optimization scheme has the form.

38 $$W_{k + 1} = W_{k} R_{{\Omega_{k} }} = W_{k} {\text{exp}}\left( { - t_{opt} \Omega_{k} } \right)$$

## Toral geometry in optimization landscape

The procedure described above requires the use of computationally expensive matrix exponentiation at each step. The decomposition of the optimization movement in the $\mathrm{U}\left(n\right)$ group into commutating rotations in complex orthogonal planes can be performed by any techniques leading to the diagonalization of the gradient matrix. The choice of Schur decomposition is dictated by the very structure of this type of decomposition. Schur decomposition produces a triangular matrix, which is assumed to be less computationally expensive than decomposition producing a diagonal matrix. The skew-Hermity of the gradient matrix (37) causes the Schur decomposition to produce, in this case, also a diagonal matrix, but with a lower computational cost than strict diagonalization techniques.

A well known fact is^40^

### Theorem 1

*If* $A={\mathbb{C}}^{n\times n}$ is a normal matrix, then there exists a unitary matrix $Q={\mathbb{C}}^{n\times n}$ such that

39 $${Q}^{H}AQ=\left[\begin{array}{cc}\begin{array}{cc}{\lambda }_{1}& 0\\ 0& {\lambda }_{2}\end{array}& \begin{array}{cc}\dots & 0\\ \dots & 0\end{array}\\ \begin{array}{cc}\vdots & \vdots \\ 0& 0\end{array}& \begin{array}{cc}\ddots & \vdots \\ \dots & {\lambda }_{m}\end{array}\end{array}\right]$$

is diagonal and each ${\lambda }_{i}$ are complex eigenvalues of $A$.

### Corollary 1

Unitary, Hermitian and skew-Hermitian matrices are normal and therefore diagonalizable.

Due to, that all skew-Hermitian matrices have purely imaginary eigenvalues ${\lambda }_{i}=i{\varphi }_{i}$, the gradient matrix $\Omega$ represented in (37) can be decomposed in the form

40 $$\Omega =UB{U}^{H}$$

where $B=\mathrm{diag}(i{\varphi }_{1},\dots ,i{\varphi }_{m})$ is a matrix with imaginary eigenvalues on the main diagonal and zeros elsewhere and $U\in \mathrm{U}(n)$. Because the matrix $B$ can be decomposed in the form $B={B}_{1}+\dots +{B}_{n}$ where ${B}_{i}=\mathrm{diag}(0,\dots ,i{\varphi }_{i},\dots ,0)$, therefore, $W=\mathrm{exp}\Omega$ can be decomposed into the commutative mutually orthogonal rotations ${W}_{i}$

41 $$W=U\mathrm{exp}\left(U\left({B}_{1}+\dots +{B}_{n}\right){U}^{H}\right)={\prod }_{i}U\mathrm{diag}\left(1,\dots ,1,{R}_{i},1,\dots ,1\right){U}^{H}={W}_{1}\cdot \dots \cdot {W}_{n}$$

where $${R}_{i}={e}^{i{\varphi }_{i}}=\mathit{cos}{\varphi }_{i}+i\mathit{si}n{\varphi }_{i}$$ and $${W}_{i}=U\mathrm{diag}(1,\dots ,1,{R}_{i},1,\dots ,1){U}^{H}.$$

In this case, exponential of the gradient matrix $\Omega$ is reduced to a simple and inexpensive calculation of function $sin{\varphi }_{i}$ and $cos{\varphi }_{i}$, which reduces the complexity of the optimization algorithm. The rotation matrices ${W}_{i}$ commute ${[W}_{i},{W}_{j}]=0$ for $i\ne j$, so the optimization process can be performed in Lie algebra $\mathfrak{u}\left(n\right)$.

In the special case of $\mathrm{U}(2)$, a diagonal form of the skew-Hermitian matrix can be expressed as

42 $$\Omega =U\left(\begin{array}{cc}i{\varphi }_{1}& 0\\ 0& i{\varphi }_{2}\end{array}\right){U}^{H}$$

Hence, the unitary matrix $W$ has the form

43 $$W=\mathrm{exp}\Omega =U\left(\begin{array}{cc}\mathrm{cos}{\varphi }_{1}+i\mathrm{sin}{\varphi }_{1}& 0\\ 0& \mathrm{cos}{\varphi }_{2}+i\mathrm{sin}{\varphi }_{2}\end{array}\right){U}^{H}$$

**Figure 2:**
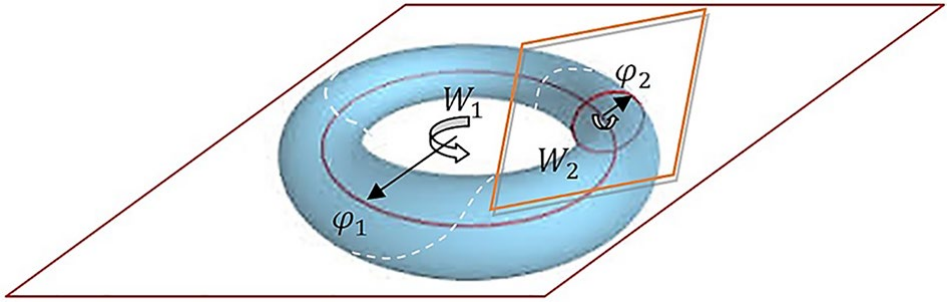
Visual representation of the toral subalgebra $\mathfrak{t}(p)$ for $p=2$ planes of rotations. The broken line marks the search curve*.* The matrices ${W}_{1}$, ${W}_{2}$, are defined as in (41).

This matrix represents the rotations in two mutually orthogonal complex plane and can be viewed as the geometry of the familiar doughnut shape, i.e., the toral geometry (Fig. 2).

This concept can be naturally transferred to the case of $\mathrm{U}(n)$ for $n>2$. The rotation matrix $W$ expressed in the form (41) represents optimization motion in one-parameter subgroup $\mathrm{exp}(t\Omega )$ as the rotation in *n*-mutually orthogonal complex plane. The commuting matrices ${W}_{i}$ represent this independent complex plane of rotations and it geometrically coincides with *n*-dimensional (maximal) torus in $\mathrm{U}(n)$. The optimization procedure based on this concept consists of translating a skew-Hermitian gradient matrix $\Omega \in \mathfrak{u}\left(n\right)$ to the diagonal form, thereby establishing the toral subalgebra. The new rotation matrix $W$ is evaluated by determining *n* values of $sin$ and $cos$ function which correspond to *n* planes of rotations. Using some line-search procedure, the optimal step size ${t}_{opt}$ is found which minimizes the cost function and then the new skew-Hermitian gradient matrix $\Omega^{\prime}$ is determined and again translated to the diagonal form which establishes the new toral subalgebra. This procedure is repeated until convergence.

## Computational complexity

In this section, the computational complexity of the proposed ICA algorithm is examined and compared with the complexity of other popular ICA algorithms in the case of $n\times m$ MIMO system. Most ICA algorithms consist of two main steps: the pre-processing stage including centering and whitening and a second stage being an optimization loop. Whitening stage includes the covariance matrix calculation, the eigenvalue decomposition (ED) and the computation of whitened signals, where the ED dominates the computational complexity through the entire pre-processing stage. Computational complexity of complex whitened process with ED calculation is equal to $2N{m}^{2}+\frac{16}{3}{m}^{3}+4Nnm$ flops, where in the estimation we made the assumption that 1 complex multiplication is equal to 4 real floating point operations^41^. The optimization stage is primarily an iterative loop consisting mainly of calculating the gradient matrix of the cost function and updating the separation matrix via matrix exponential. There are many alternative methods to accurately and approximately matrix exponential^39^. In the simulation the Matlab's expm function was used that uses one of the most efficient methods, i.e., the diagonal $(q,q)$-Padé approximation with scaling and squaring^42^. This approximation, in real case requires about $\left(q+j+\frac{1}{3}\right){m}^{3}$ flops to evaluate, where $j$ denotes the scaling factor^39^. In typical applications and in general matrix it is estimated between $20 {m}^{3}$ and $30{\mathrm{ m}}^{3}$ and for skew-symmetric matrices around $10 {m}^{3}$^43,44^. Moler and Van Loan in^39^ estimate the complexity of real Schur decomposition to be around ${5m}^{3}$ based on the complexity of the QR decomposition used to create the similarity eigenvectors matrix. By using the most computationally efficient and numerically stable approach for QR decomposition, i.e., the modified Gram-Schmidt procedure the complexity cost can be reduced to ${2m}^{3}$^45^. The computational complexity of the individual stages of the proposed algorithm is presented in Table 1, where we estimate the complexity of the matrix exponential as $4\cdot 2{m}^{3}$.

**Table 1:** Numerical complexity of the proposed algorithm (in complex case).

Stages	Numerical complexity (flops)
Preprocessing (centering and whitening)	${\mathrm{C}}_{\mathrm{w}}=2N{m}^{2}+\frac{16}{3}{m}^{3}+4Nnm$
Optimization (loop)	
Cost function evaluation	$8N{m}^{2}$
Gradient matrix estimation	$8N{m}^{2}$
Skew-Hermitization of gradient matrix	$8{m}^{3}$
Exponentiation	$8{m}^{3}$
Update of *W*	$4{m}^{3}$

Based on this analysis, the computational cost of geodesic complex ICA algorithm with diagonal $(q,q)$-Padé approximation using Matlab's expm function can be estimated as ${\mathrm{C}}_{\mathrm{w}}+\left(16N{m}^{2}+12{m}^{3}+4\left(q+j+\frac{1}{3}\right){m}^{3}\right)it$ flops, where ${\mathrm{C}}_{\mathrm{w}}$ is the complexity of preprocessing stage, and $it$ is the number of iterations of optimization loop. The computational cost of the proposed geodesic complex ICA algorithm with Schur decomposition can be estimated as ${\mathrm{C}}_{\mathrm{w}}+\left(16N{m}^{2}+12{m}^{3}+{8m}^{3}\right)it$ flops.

The computational complexity of the classic Infomax algorithm used in our experiment (section "MIMO systems' simulation results") is equal to ${\mathrm{C}}_{\mathrm{w}}+4\left({m}^{2}+{m}^{3}+4m+5Nm\right)it$, where $N$ is the number of data samples. The computational cost of fixed-point process (24) is $12{m}^{2}N$ so the numerical complexity of symmetric complex FastICA algorithm is equal to ${\mathrm{C}}_{\mathrm{w}}+4\left({n}^{3}+\left(\frac{16{m}^{3}}{3}+ {m}^{2}+3N{m}^{2}\right)it\right)$ flops^41^. In Jacobi-like algorithms such as JADE the second stage is usually joint orthogonal diagonalization of the set of $M$ matrices of cummulants of the size $m\times m$. Its numerical complexity is equal to $2Im\left(m-1\right)(4Mm+17M+4m+75)$ flops, where $I$ stands for the number of executed sweeps and $M$ is the number of matrices to be jointly diagonalized. Computational complexity of JADE algorithm requiring computation of $2q$-th order cumulant of $m$-dimensional random process which is equal to ${\mathrm{C}}_{\mathrm{w}}+12N{f}_{4}\left(m\right)+12N{m}^{2}+min\left(\frac{16{m}^{6}}{3},24{m}^{3}\left({m}^{2}+3\right)+2Im\left(m-1\right)(75+21m+4{m}^{2})\right)$ flops (in complex case) where ${f}_{4}\left(m\right)$ denotes the number of free entries of the cumulant array and is given as a function of $m$. A detailed analysis of the computational complexity of popular ICA algorithms can be found in^41^.

The results of the computational complexity analysis of the algorithms used in the simulation are presented in Table 2.

**Table 2:** Comparison of the computational complexity of the ICA algorithms used in the simulation.

ICA algorithm	Numerical complexity (flops)
Proposed Algorithm with Schur decomposition	${\mathrm{C}}_{\mathrm{w}}+\left(16N{m}^{2}+12{m}^{3}+{8m}^{3}\right)it$
Proposed Algorithm with expm	${\mathrm{C}}_{\mathrm{w}}+\left(16N{m}^{2}+12{m}^{3}+4\left(q+j+\frac{1}{3}\right){m}^{3}\right)it$
Infomax	${\mathrm{C}}_{\mathrm{w}}+4\left({m}^{3}+{m}^{2}+4m+5Nm\right)it$
JADE	${\mathrm{C}}_{\mathrm{w}}+12N{f}_{4}\left(m\right)+12N{m}^{2}+min\left(\frac{16{m}^{6}}{3},24{m}^{3}\left({m}^{2}+3\right)+2Im\left(m-1\right)(75+21m+4{m}^{2})\right)$
cFastICA	${\mathrm{C}}_{\mathrm{w}}+4\left({n}^{3}+\left(\frac{16{m}^{3}}{3}+ {m}^{2}+3N{m}^{2}\right)it\right)$

Assuming that $N>>m$ the leading component (per iteration) in the estimates shown in Table 2 is of the order of $O(N{m}^{2})$. In the case of the proposed ICA algorithm, this component is $16N{m}^{2}$ using both (in the exponentiation step) the Schur decomposition and the expm function. The difference is only in the computational cost of the exponentiation operation, which is $8{m}^{3}$ in the former case and about $40{m}^{3}$ in the latter. In the case of the Infomax algorithm, the leading component is $20Nm$, however, it should be noted that the convergence of this type of classical gradient algorithms is achieved with a much higher number of iterations $it$, so the total computational cost is higher than for the other algorithms. In the case of the JADE algorithm, the leading component is $12N{m}^{2}$ and it is smaller than in the proposed algorithm however, the second leading component is even up to the order of $O\left({m}^{6}\right)$, therefore, its computational complexity strongly depends on the number of transmitted signals $m$, which is a well-known drawback of algebraic ICA algorithms. The computational cost of the cFastICA algorithm is less than the proposed one and its two leading components are $12N{m}^{2}+(48{m}^{3})/3$ however, as shown in ^46^, this algorithm with the number of transmitted signals $m\ge 6$ is characterized by instability and non-convergence, which were not observed in the case of the proposed approach.

## Multiple-Input Multiple-Output Systems

Multiple antenna techniques are used to improve the performance and robustness of wireless links in the telecommunication field. The Multiple-Input Multiple-Output system uses an array of antennas for both transmitting and receiving end. This system gives higher data rate, higher transmit and receive diversity through spatial multiplexing in wireless communication systems^47^. Wireless communication is currently facing a lot of problems due to increasing levels of interference. Multipath is a propagation phenomenon in which radio signals reach the receiving antenna along two or more paths. The main causes of multipath are atmospheric, dispersion, reflection from water bodies and ground objects such as mountains and buildings. Delayed signals are the results of reflection from terrain features such as trees, buildings or hills. These delayed waves interfere with the direct wave and causes interference that degrades network performance. In radio communication systems, i.e. GSM multipath can cause errors for example inter-symbol interference and affect the quality of communication. Copies of the signal coming via different paths can undergo different attenuation, distortions, delays and phase shift. This causes harmful interference which can in turn significantly reduce the strength of the received signal. This phenomenon is called fading. Fading is usually modeled as a time-varying random change in the amplitude and phase of the transmitted signal. There are several basic fading models such as Weibull, Nakagami, Rician, Log-Normal Shadowing or Rayleigh fading model^48^. The last model is quite reasonable for scattering mechanisms where there are many small reflections.

The time invariant MIMO channel can be described similarly to the noisy ICA model (2)

44 $$y = Hx + \vartheta$$

where $x\in {\mathbb{C}}^{{n}_{t}}$, $y\in {\mathbb{C}}^{{m}_{r}}$ and $\vartheta \sim CN(0,{{\sigma }^{2}I}_{{n}_{t}})$ denoted the transmitted signal, received signal and circular symmetric Gaussian noise vector with zero mean and covariance matrix ${{\sigma }^{2}I}_{{n}_{t}}$, respectively. The ${n}_{t}$ and ${m}_{r}$ denoted the number of transmitting and receiving antennas and $H\in {\mathbb{C}}^{{n}_{t}\times {m}_{r}}$ is the channel matrix. Element ${h}_{ij}$ of the channel matrix $H$ is the channel gain from transmitting antenna $j$ to receiving antenna $i.$

A very common MIMO fading model is the independent identically distribute (i.i.d.) Rayleigh fading model where entries of the channel gain matrix $H$ are i.i.d. and circular symmetric complex Gaussian. Channel taps are independent random coefficients with power distributed according to Rayleigh distribution.

## MIMO systems' simulation results

**Figure 3:**
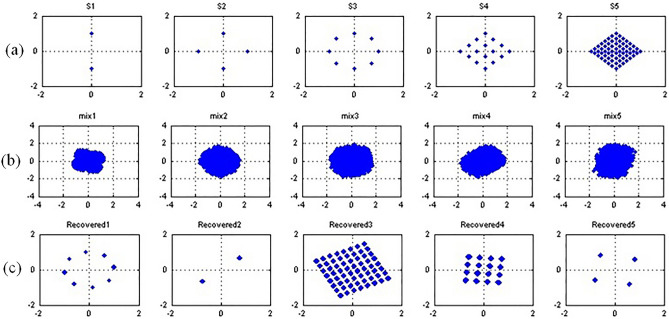
The constellation patterns corresponding to (**a**) five transmitted signals, (**b**) five of the eight received signals and (**c**) the five recovered signals using proposed algorithm.

In this section the proposed algorithm was used for separation independent signals in MIMO system. The choice of the research facility in the form of the MIMO system was dictated by the ease of interpretation and evaluation of the results of the cICA algorithms. The graphical presentation of the results as in Fig. 3 allows for a quick evaluation of the signal separation results, which in other cases is not always easy and possible. In addition, the experiment was to show an example of the use of the proposed cICA algorithm in on-line applications where the speed of algorithms is of key issues. In the case of MIMO systems, other solutions such as coherent decomposition are used with great success. However, it should be emphasized that the purpose of the experiment was not to compare the proposed separation algorithm with the solutions used in MIMO systems, but only to compare it with other cICA-type algorithms in the context of mixed complex signals from a real system.

The proposed algorithm (denoted as Schur) was compared with the same algorithm but using exponential map (denoted as Exp) and with the classical Euclidean gradient descent algorithm (denoted as Euclid) which enforces unitarity of $W$ after every iterations and with the Euclidean gradient algorithm with extra penalty term (denoted as EuclidPen). In addition, the cFastICA and complex JADE algorithms were tested, as representatives of quasi-Newton and algebraic ICA algorithms, respectively. The standard Matlab expm function was used in the Exp algorithm to map from Lie algebra $\mathfrak{u}\left(n\right)$ to Lie group $\mathrm{U}\left(n\right)$.

The simulation uses the classic form of the cost function based on the negentropy approximation expressed in (12) and presented in Section "Lie group and their Lie algebra". The nonlinear function was chosen in the form $\mathrm{G}\left(u\right)=\frac{1}{2}{u}^{2}, u={\left|Wx\right|}^{2}$. The choice of such non-linearity was made on the basis of^46^, where the high efficiency of the application of such non-linearity in the algorithms of blind separation of digitally modulated signals was demonstrated.

A number of ${n}_{t}$ independent zero-mean signals are sent by using ${n}_{t}$ antennas and they are received by ${m}_{r}$ receiving antennas. In the experiment a frequency flat MIMO channel matrix $H$ with the Rayleigh fading and AWGN model was used. The case of a symmetrical MIMO channel with ${n}_{t}={m}_{r}=n$ sources and a non-symmetrical MIMO channel with ${n}_{t}<{m}_{r}$ was simulated. In the first case, the operation of the algorithms was tested with the use of a different number of transmitted signals from $n=2$ to $n=8$. In the case of a non-symmetrical MIMO channel, the cases ${n}_{t}\times {m}_{r}=2 \times 3$, $3 \times 5$ and $5 \times 8$ were tested. The $N=5000$ samples of the phase shifted keying signals, binary BPSK and PSK(8), quadrature amplitude modulated signals QAM(4), QAM(16) and QAM(64) were used. Figure 3a and b show the transmitted and received signals, respectively.

The software was implemented in Matlab 7.9 on a PC (Intel i7 2.8 GHz CPU, 8 GB RAM). An example of the simulation result using proposed algorithm is shown in Fig. 3c. In the line search stage an adaptive step-size selection procedure was used with bracketing and selection phases (B-S) as in^49^ with complex strong Wolf conditions and quadratic interpolation of the cost function. The experiment showed, however, despite of the small number of iterations, large convergence times of the algorithm which proves the high computational cost of multiple evaluation of cost function in B-S stage. So, the optimal step size $\mu$ was chosen experimentally, as in^46^, and the rest of the experiment was carried out for the fixed $\mu =0.5$. The convergence criterion was used as in the cFastICA algorithm^23^ denoted as F criterion. The M criterion which additionally takes into account the convergence of the cost function was also alternatively used. The F criterion is a measure of $W$ deviation from unitarity, while in M criterion an additional condition of cost function convergence has been introduced in the form: if ${J}_{k}-{J}_{k-1}<{\varepsilon =10}^{-4}$ break.

In performance analysis, performance indicators, both classic for ICA methods and those with the application context, were taken into account. In the first part of the experiment, low-power noise was used, i.e. the signal to noise ratio was *SNR* = 30 dB. Performance of separation was measured by classical parameter more suitable for the noiseless ICA model, i.e. Amari Performace Index (API) defined as^33^

45 $$\mathrm{API}=\sum_{i=1}^{n}\left(\sum_{j=1}^{n}\frac{\left|{p}_{ij}\right|}{{\mathrm{max}}_{k}\left|{p}_{ik}\right|}-1\right)+\sum_{j=1}^{n}\left(\sum_{i=1}^{n}\frac{\left|{p}_{ij}\right|}{{\mathrm{max}}_{k}\left|{p}_{kj}\right|}-1\right)$$

where ${p}_{ij}$ is $\left(i,j\right)$-th element of the global system matrix $P=W{A}^{-1}$. In the second part of the experiment, an analysis of the separation quality at higher and various noise powers was carried out. In this case, an adequate performance indicator is the signal-to-interference-plus-noise ratio (SINR), for the *k*-th estimated signal with unit variance, defined by^50^

46 $${\mathrm{SINR}}_{\mathrm{k}}=\frac{{\left(WA\right)}_{kk}^{2}}{{\sum }_{i\ne k}^{d}{\left(WA\right)}_{ki}^{2}+{\sigma }^{2}{\sum }_{i=1}^{d}{W}_{ki}^{2}}$$

Traditionally, performance gain in MIMO systems is measured by bit error rate (BER). Performance analysis was therefore supplemented with an analysis of the impact of the SNR on the BER parameter in the case of AWGN and Rayleigh fading channel. The analysis of SINR and BER required removing the ambiguity of order, scale and sign of separated signals, which is inherent in all ICA methods. In addition, the BER analysis also required removing the phase ambiguity of the separated signals.

**Figure 4:**
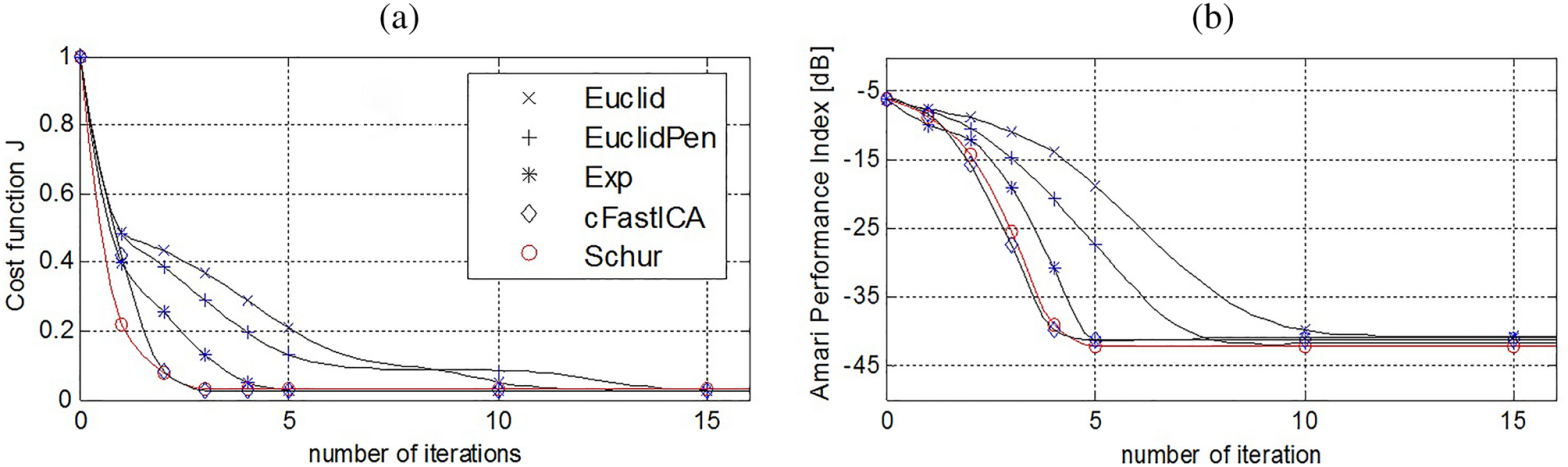
Examples of the evolution of the cost function (12) (**a**) and Amari Performance Index (**b**) for different algorithms used in the simulation.

**Figure 5:**
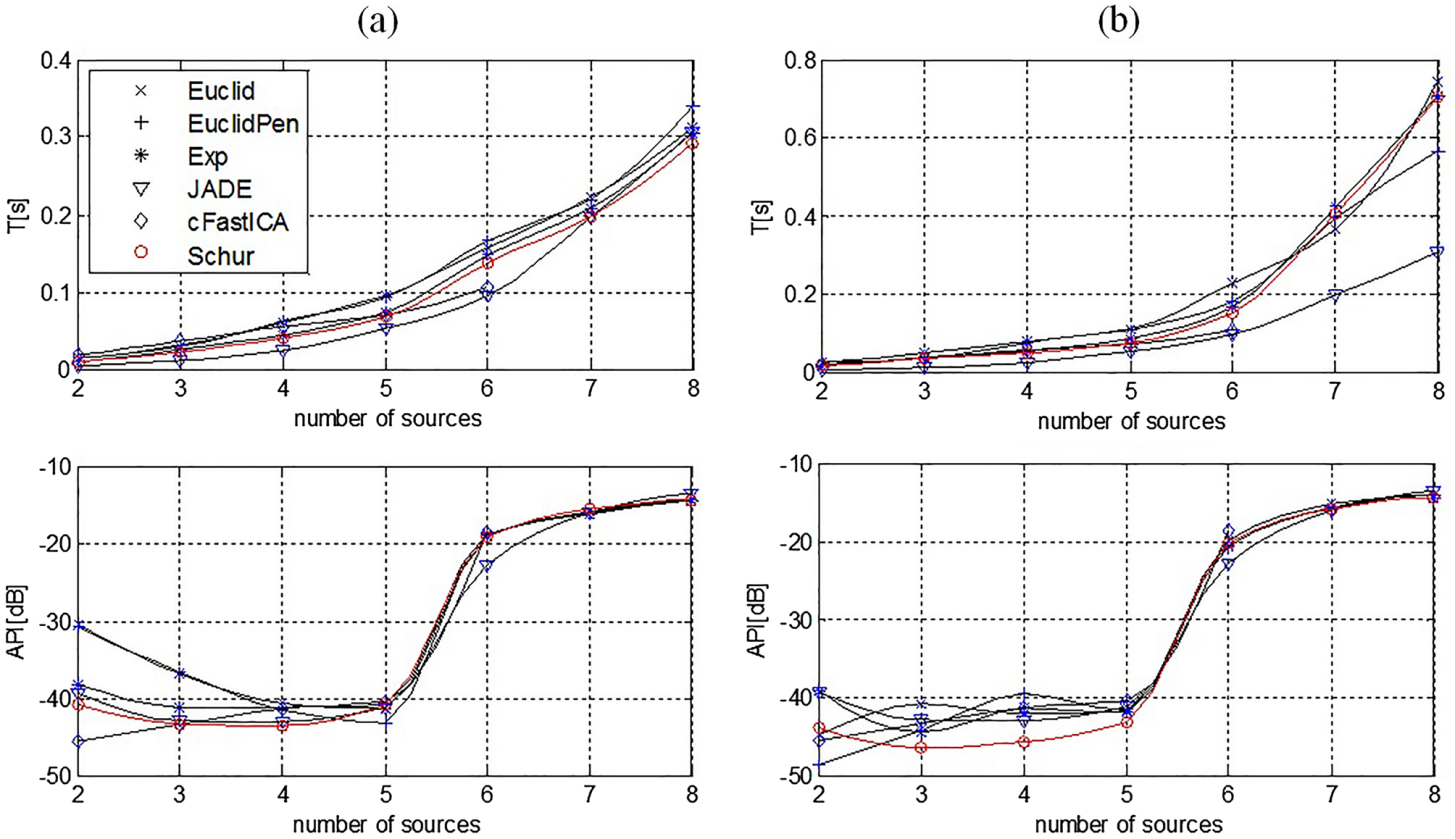
Comparison of the average convergence times and API of the tested algorithms for (**a**) M and (**b**) F criterion, respectively versus number of sources in symmetrical MIMO system.

The results of running time of the algorithms and API were averaged over 100 trials. Examples of evolution of the cost function and API versus the number of iterations are presented in Fig. 4a and b, respectively. The results of the obtained averaged convergence times and API relative to the number of transmitted signals for tested proposed algorithm and of comparison with classic SD, cFastICA and complex JADE in application to symmetric MIMO system are presented in Fig. 5a and b for M and F criterion, respectively.

First of all, it should be noted that in general the convergence times of ICA algorithms in the case of the simulated MIMO channel are longer than in the case of the linear complex ICA problem presented in^46^, which proves the greater complexity of the simulated problem.

For the symmetrical MIMO channel, shorter convergence times of the algorithms while maintaining good separation quality (i.e., API) were obtained using the M convergence criterion (Fig. 5a). In the case of a symmetrical MIMO channel and for $n<5$, the highest convergence speed was achieved for the JADE algorithm. The proposed Schur algorithm had the highest convergence speed among the tested algorithms for $n\ge 7$ signals (speed increase of about 5.3% over JADE algorithm). The cFastICA algorithm gave good results in terms of convergence speed and separation quality for $n<6$. For the number of transmitted signals $n\ge 6$, cFastICA did not converge in the set maximum number of iterations, i.e., 200 iterations. The Euclid and EuclidPen algorithms do not operate in the appropriate parameter space, so the convergence speed was lower compared to the other tested algorithms.

**Figure 6:**
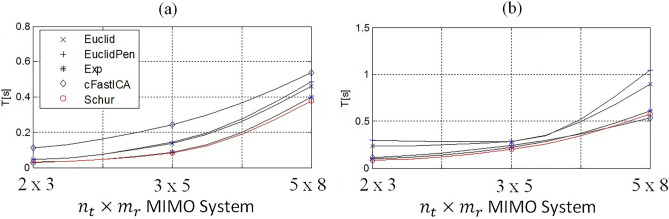
Comparison of the average convergence times of the tested algorithms for (**a**) M and (**b**) F criterion, respectively versus type of non-symmetrical MIMO system.

In the case of a non-symmetrical MIMO channel, the separation quality using the M criterion with the parameter ${\varepsilon =10}^{-4}$ was not sufficient. Therefore, the value of this parameter was reduced to the level ${\varepsilon =10}^{-5}$, which ensured a similar quality of signal separation as in the symmetrical case. The results of the obtained averaged convergence times relative to the type of MIMO channel for tested algorithms in application to non-symmetric MIMO system are presented in Fig. 6a and b for M and F criterion, respectively. It should be noted that in the non-symmetrical case, the JADE algorithm completely failed in each of the tested MIMO channel types. The cFastICA algorithm worked correctly and the convergence speed was the highest when using the F criterion for the $5 \times 8$ MIMO channel. In the case of $2 \times 3$ and $3 \times 5$, the fastest convergence was noted for the proposed Schur algorithm. When using the M criterion, the convergence speed of all tested algorithms was higher than that of cFastICA, and the highest speed in each of the tested non-symmetrical MIMO channel types was achieved by the proposed Schur algorithm (Fig. 6a).

**Figure 7:**
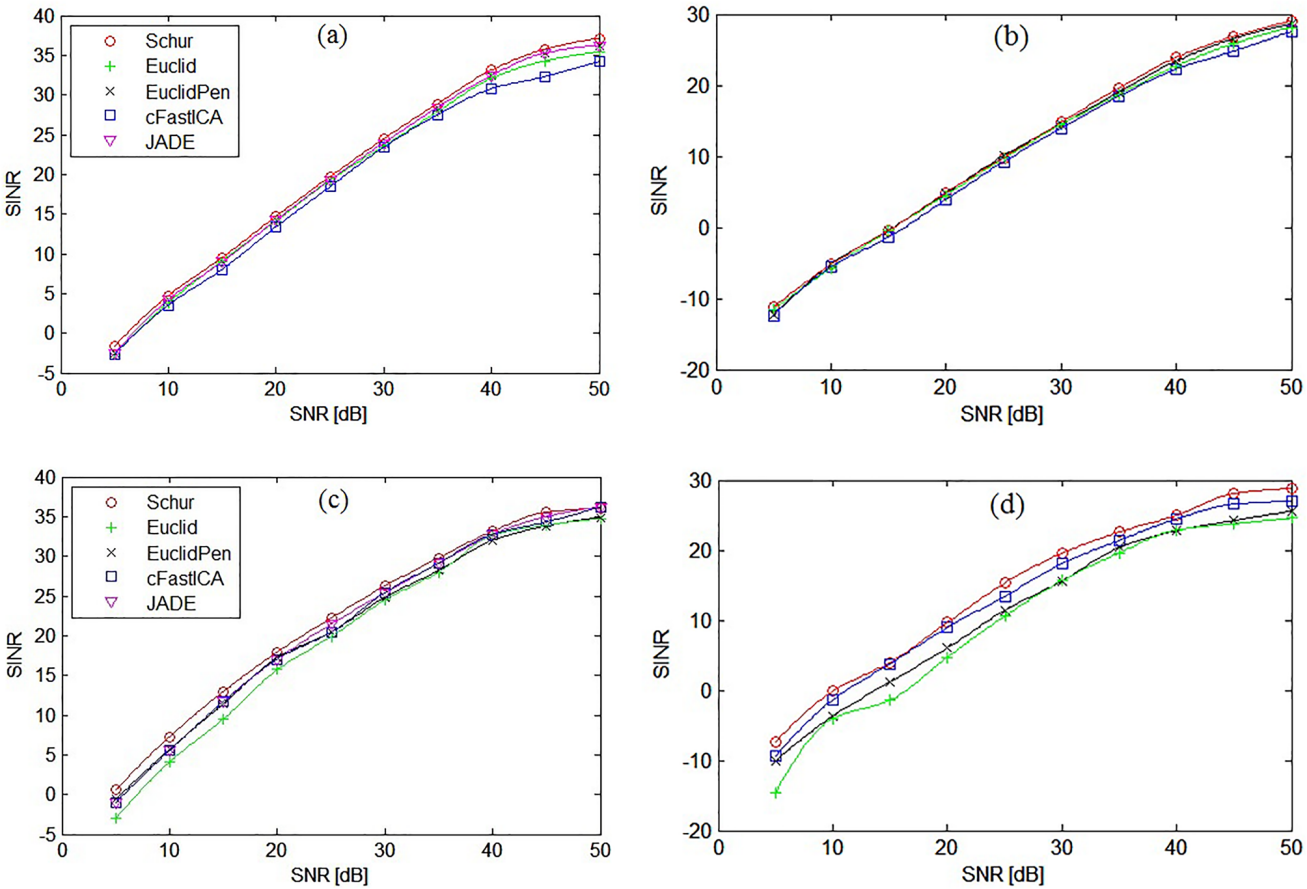
SINR performance comparison of ICA algorithms for: (**a**) symmetrical 3 × 3 MIMO system and AWGN channel, (**b**) non-symmetrical 5 × 8 MIMO system and AWGN channel, (**c**) symmetrical 3 × 3 MIMO system and Rayleigh channel, (**d**) non-symmetrical 5 × 8 MIMO system and Rayleigh channel.

**Figure 8:**
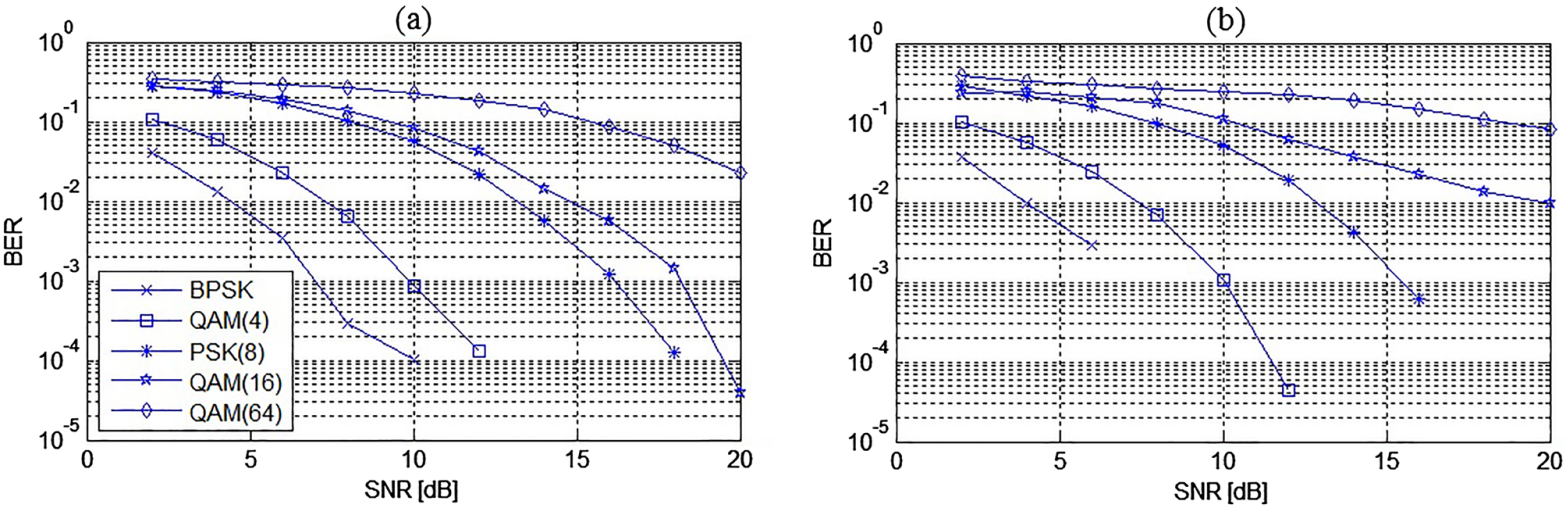
BER performance of separated signals in (**a**) AWGN and (**b**) Rayleigh fading channel.

**Figure 9:**
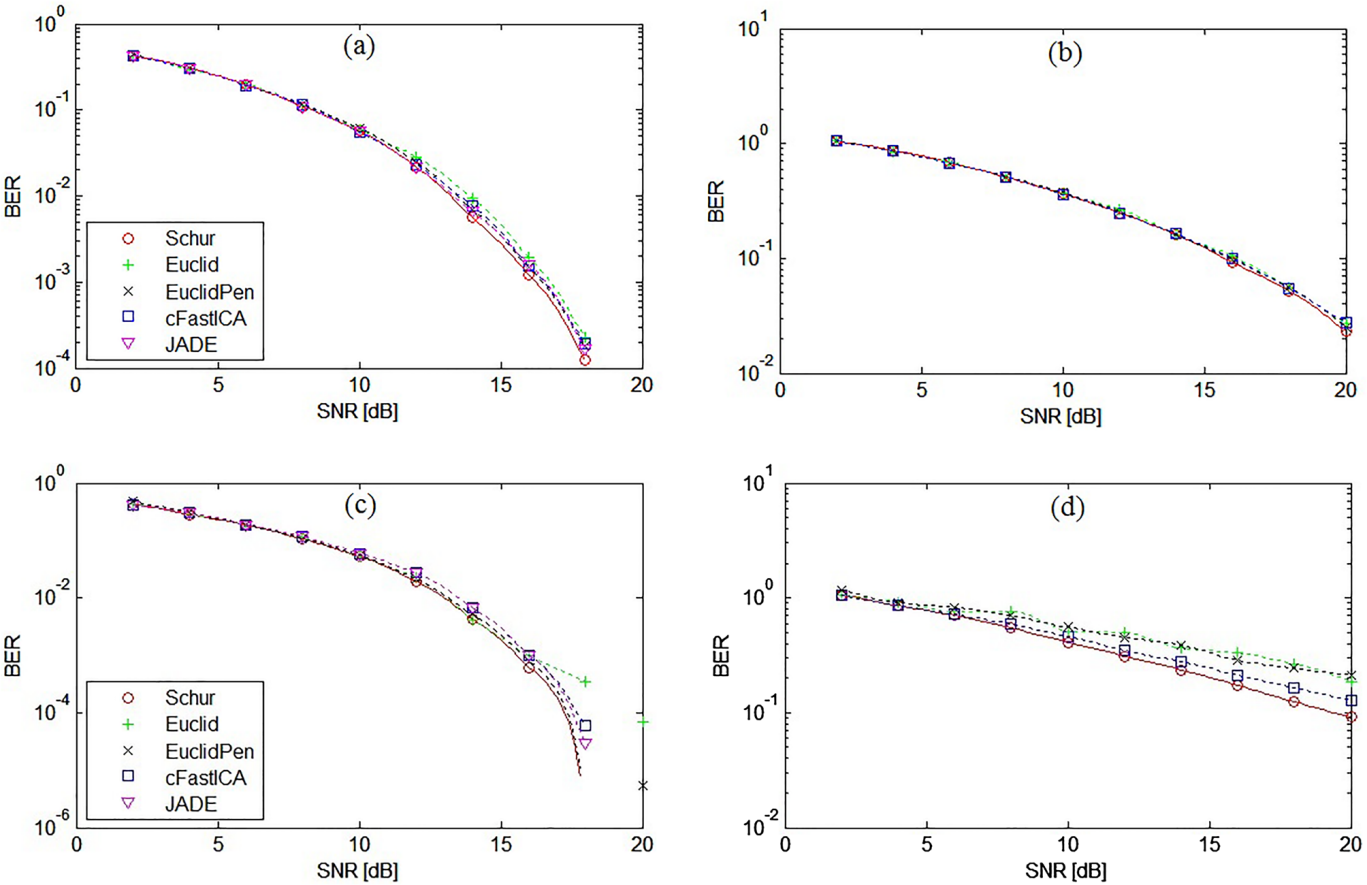
Average BER performance comparison of ICA algorithms for: (**a**) symmetrical 3 × 3 MIMO system and AWGN channel, (**b**) non-symmetrical 5 × 8 MIMO system and AWGN channel, (**c**) symmetrical 3 × 3 MIMO system and Rayleigh channel, (**d**) non-symmetrical 5 × 8 MIMO system and Rayleigh channel.

Figures 7 show a comparative analysis of the SINR vs SNR parameter for all tested algorithms for the symmetrical 3 × 3 and non-symmetrical 5 × 8 MIMO system with AWGN and Rayleigh fading channel. The figures do not show the results for the Exp algorithm due to the fact that the results were identical to those obtained for the proposed Schur algorithm. Figure 8 shows the obtained BER values for each of the extracted signals separately for the case of the non-symmetrical 5 × 8 system. As could be expected, the largest error was obtained for signals with the most complicated constellation scheme, i.e. for QAM(16) and QAM(64) signals, which was also reflected in the results of the average BER value presented in Figs. 9.

Figure 9a was obtained for AWGN channel and the symmetrical 3 × 3 system where the BPSK, QAM(4) and PSK(8) signals were used, while Fig. 9b for the non-symmetrical 5 × 8 system where the QAM(16) and QAM(64) signals were additionally transmitted. As can be seen from the presented analysis of SINR and BER parameters, the proposed method is characterized by the highest separation quality parameters among the tested ICA algorithms for both symmetrical and non-symmetrical MIMO systems.

Based on the results of the experiment, it was found that the convergence speed of the proposed Schur algorithm in the case of a symmetrical MIMO channel is the highest among the tested algorithms in the case of a larger number of transmitted signals ($n\ge 7$). The quality of separation (API, BER and SINR) in this case of all tested algorithms was similar as shown in Figs. 5, 7 and 9. The proposed algorithm performed particularly well in the non-symmetric case. In each of the tested cases with M criterion the greatest convergence speed was achieved. The highest SINR result (Fig. 7) and the lowest BER result (Fig. 9). The average increase in convergence speed compared to the cFastICA algorithm was 56% and 25% compared to the other gradient algorithms (Fig. 6). It should be noted that the experiment also showed an increase in the speed of the exponentiation operation using toral decomposition of the gradient matrix compared to the standard Matlab expm procedure. The average increase in the speed of the Schur algorithm was 7% (6,6% for symmetrical and 7,8% for non-symmetrical case), which proves the lower computational cost of the proposed exponentiation procedure.

An important observation is that the classical representatives of ICA algorithms from the Newton and algebraic groups, i.e. cFastICA and complex JADE, which worked well in the case of linearly mixed signals^46^, failed in the case of the symmetrical (and large number of signals) and non-symmetrical MIMO systems, respectively. The gradient flow algorithms Exp and the proposed Schur in each of the tested cases gave good results both in terms of separation quality and speed, which proves the high universality of the proposed method and potential practical use in on-line applications.

## Conclusion

In this paper, geodesic flow optimization algorithm with toral decomposition have been introduced and tested in the MIMO telecommunication system. This type of decomposition establishes in Lie algebra $\mathfrak{u}\left(n\right)$ a toral subalgebra $\mathfrak{t}(p)$, which allows for low computationally-expensive mapping to Lie group $\mathrm{U}\left(n\right)$. The theoretical part of this paper presents Riemann and Lie structure of the group $\mathrm{U}\left(n\right)$. The method of introducing the toral subalgebra $\mathfrak{t}(p)$ using the diagonalization of skew-Hermitian gradient matrix is also presented. In the experimental part, the proposed algorithm was tested in the MIMO system and its operation was compared with other SD algorithms, as well as with the classic cFastICA and JADE algorithms. The simulation showed good results in terms of speed of operation and quality of separation, especially in an nonsymmetrical MIMO system. The experiment showed that the proposed method is a high-performance, low-cost, and therefore, fast algorithm that enables the solution of the complex ICA problem in practical application.

## Supplementary Information

Supplementary Information.

## Data Availability

The datasets used and/or analysed during the current study available from the corresponding author on reasonable request.
