# Supplementary material for: Fast gradient algorithm for complex ICA and its application to the MIMO systems
Source: Sci Rep. 2023 Jul 19;13:11633. doi: 10.1038/s41598-023-36628-w (PMC10356829; doi:10.1038/s41598-023-36628-w)
Supplement: Supplementary file 1 — Supplementary Information. [file 41598_2023_36628_MOESM1_ESM.docx]

**Appendix. Complex preliminaries**

Searching for extremes requires determining the gradient of the real-valued cost function on the complex domain. There are several definitions of the differentiation of complex functions. A complex derivative of complex function $f=u+iv:U\to\mathbb{C}$, where $U$ is an open set in $\mathbb{C}$ is defined as [51]

$\lim_{h\to0} \frac{f\left( c+h \right)-f(c)}{h}\mathbb{\in C}$ (A1)

If this limit exists and is independent of the direction in which $h$ reaches 0 on the complex plane, then the function $f$ must satisfy the Coauchy-Riemann conditions (C-R)

$\frac{\partial u}{\partial z_{R}}=\frac{\partial v}{\partial z_{I}}$ and $\frac{\partial u}{\partial z_{I}}=-\frac{\partial v}{\partial z_{R}}$ (A2)

Functions that satisfy C-R conditions throughout the domain are called analytical, holomorphic, or $\mathbb{C}$-differentiable functions. However, the C-R condition is too strong for the cost functions used in signal processing. Real valued function (i.e., $v=0$) satisfying the C-R conditions would have to be a constant function, which obviously excludes it as a cost function used in optimization. Therefore, a non-analytical function must be used there. A less restrictive definition of differentiation is real sense differentiation or $\mathbb{R}$-differentiation. Function $f=u+iv:U\to\mathbb{C}$ is $\mathbb{R}$-differentiable at $c\mathbb{\in C}$ if there exists widely linear (or $\mathbb{R}$-linear) function $L_{c}\left( h \right)=\alpha h+\beta h^{*}, \alpha,\beta\mathbb{\in C}$, such that [51]

$\lim_{\left| h \right|\to0} \frac{f\left( c+h \right)-f\left( c \right)-L_{c}(h)}{\left| h \right|}=0$ (A3)

Function $L_{c}\left( h \right)$ is called the $\mathbb{R}$-differential of $f$ at $c.$ If the function $f$ is $\mathbb{R}$-differentiable then $L_{c}\left( h \right)=\frac{\partial f}{\partial z}(c)h+\frac{\partial f}{\partial z^{*}}(c)h^{*}$, where $\frac{\partial f}{\partial z}$ and $\frac{\partial f}{\partial z^{*}}$ are complex partial derivatives defined below. If real and imaginary part of $f$ have real partial derivative at $c\mathbb{\in C}$, one defines real partial derivative of $f$ as [52]

$\frac{\partial f}{\partial x}=\frac{\partial(u+iv)}{\partial x}\triangleq\frac{\partial u}{\partial x}+i\frac{\partial v}{\partial x}$ and $\frac{\partial f}{\partial y}=\frac{\partial(u+iv)}{\partial y}\triangleq\frac{\partial u}{\partial y}+i\frac{\partial v}{\partial y}$ (A4)

These partial derivatives define the directional derivative of the function $f$ in the direction of real and imaginary axis of the complex plane, respectively. In turn, complex partial derivative of $f$ with respect to $z$ and $z^{*}$ are defined as

$\frac{\partial f}{\partial z}\triangleq\frac{1}{2}\left( \frac{\partial f}{\partial x}-i\frac{\partial f}{\partial y} \right)$ and $\frac{\partial f}{\partial z^{*}}\triangleq\frac{1}{2}\left( \frac{\partial f}{\partial x}+i\frac{\partial f}{\partial y} \right)$ (A5)

Differential operators $\frac{\partial f}{\partial z}$ and $\frac{\partial f}{\partial z^{*}}$ are the basis of the so-called Wirtinger or $\mathbb{C} \mathbb{R}$-calculus [53]. Formally, in Wirtinger calculus, the function $f$ is treated as a bivariate function $f(z,z^{*})$ treating $z$ and $z^{*}$ as independent variables. Then complex partial derivative $\frac{\partial f}{\partial z}$ and $\frac{\partial f}{\partial z^{*}}$ are determined by treating $z$ and $z^{*}$, respectively, as constant in $f$. The usefulness of this calculus lies in the fact that the principles of summation, product, gradient and chain role are formally fulfilled as for real partial derivative. In this approach, C-R conditions are presented in a simple form as $\frac{\partial f}{\partial z^{*}}=0$, which means that holomorphic functions depend only on $z$ and not on $z^{*}$. This means that in the case of holomorphic functions, the classic complex derivative in (A1) coincides with $\frac{\partial f}{\partial z}$ in (A5) and that it is a special case of the Wirtinger calculus.

At the stationary point of the real valued function $f$*,* both $\frac{\partial f}{\partial z}$ and $\frac{\partial f}{\partial z^{*}}$ vanish [51]. It is important, however, that only $\frac{\partial f}{\partial z^{*}}$, also called conjugate partial derivative, defines the direction of maximum rate of change, i.e., it defines complex gradient.

The theory of Wirtinger calculus extends naturally to the case of complex valued functions of vector or matrix domain [34]. For real differentiable functions $f :U \subset\mathbb{C}^{n}\times\mathbb{C}^{n}\mathbb{\to C}$ we define $\nabla_{z}f(z,z^{*})\equiv\frac{\partial f}{\partial z}\triangleq\left( \frac{\partial f}{\partial z_{1}},\ldots,\frac{\partial f}{\partial z_{n}} \right)^{T}$ for $z={(z_{1,\ldots,}z_{n})}^{T}$ and similarly $\nabla_{z^{*}}f(z,z^{*})\equiv\frac{\partial f}{\partial z^{*}}\triangleq\left( \frac{\partial f}{\partial z_{1}^{*}},\ldots,\frac{\partial f}{\partial z_{n}^{*}} \right)^{T}$, where $\frac{\partial f}{\partial z_{i}}$ and $\frac{\partial f}{\partial z_{i}^{*}}$ are defined in (5). Similarly for complex valued function $f$ of matrix argument $Z=\left( z_{ij} \right)$ we can define $\nabla_{Z}f\equiv\frac{\partial f}{\partial Z}\triangleq\left( \frac{\partial f}{\partial z_{ij}} \right)^{T}$ and $\nabla_{Z^{*}}f\equiv\frac{\partial f}{\partial Z^{*}}\triangleq\left( \frac{\partial f}{\partial z_{ij}^{*}} \right)^{T}$ as $n\times m$ complex matrix where $\frac{\partial f}{\partial z_{ij}}$ and $\frac{\partial f}{\partial z_{ij}^{*}}$ are defined in (5). For real valued function $f$ at the stationary point both gradient $\nabla_{z}f$ (or $\nabla_{Z}f$ in matrix case) and conjugate gradient $\nabla_{z^{*}}f$ (or $\nabla_{Z^{*}}f$ in matrix case) also vanish. Hence gradient descent optimization scheme of $f$ use the following update role $\Delta z=z_{t+1}-z_{t}=-\mu\nabla_{z^{*}}f$ for vector case and

$\Delta Z=Z_{t+1}-Z_{t}=-\mu\nabla_{z^{*}}f$ (A6)

in matrix case. Similarly in this case the direction of maximum rate of change of function $f$ is determined by $\nabla_{z^{*}}f$ (or $\nabla_{Z^{*}}f$ in matrix case), i.e., by conjugate gradient.

Expressing $f\left( z \right)$ (or $f\left( Z \right)$ in matrix case) in dual form $f\left( z,z^{*} \right)$ (or $f\left( Z,Z^{*} \right)$ in matrix case) and defining vector and matrix inner product as $\left\langle u,w \right\rangle=u^{H}w$ and $\left\langle U,W \right\rangle=trace(U^{H}W)$ respectively, it can be written first-order Taylor expansion in terms of the both arguments. In vector case

$\Delta f\left( z,z^{*} \right)=\left\langle\Delta z, \nabla_{z^{*}}f \right\rangle+\left\langle\Delta z^{*}, \nabla_{z}f \right\rangle=2\mathcal{R}e\{\left\langle\Delta z, \nabla_{z^{*}}f \right\rangle\}$ (A7)

and similarly in matrix case

$\Delta f\left( Z,Z^{*} \right)=\left\langle\Delta Z, \nabla_{Z^{*}}f \right\rangle+\left\langle\Delta Z^{*}, \nabla_{Z}f \right\rangle=2\mathcal{R}e\{\left\langle\Delta Z, \nabla_{Z^{*}}f \right\rangle\}$ (A8)

Update role of the form (A7) and (A8) leads to a nonpositive increment $\Delta f=-2\mu\left\| \nabla_{Z}f \right\|^{2}$, that can be easily shown using Couchy-Schwartz inequality [51].
